# Cross-section analysis of coal workers’ pneumoconiosis and higher brachial-ankle pulse wave velocity within Kailuan study

**DOI:** 10.1186/s12889-017-4048-7

**Published:** 2017-02-02

**Authors:** Yao Zheng, Lirong Liang, Tianbang Qin, Guang Yang, Shasha An, Yang Wang, Zhifang Li, Zhongda Shao, Xiuping Zhu, Taicheng Yao, Shouling Wu, Jun Cai

**Affiliations:** 1 Kailuan Hospital, Tangshan, Hebei China; 2 Beijing Institute of Respiratory Medicine, Beijing Chaoyang Hospital, Capital Medical University, Beijing, China; 3 Occupational Disease Prevention and Treatment Hospital of Kailuan Colliery Group, Tangshan, Hebei China; 4 Department of Cardiology, Chaoyang Hospital, Capital Medical University, Beijing, China

**Keywords:** Silica dust, Coal workers’ pneumoconiosis (CWP), Arterial stiffness, Brachial-ankle pulse wave velocity

## Abstract

**Background:**

Brachial-ankle pulse wave velocity (baPWV) is an independent predictor of cardiovascular events and mortality. However, there is no related data on the association of baPWVwith coal workers’ pneumoconiosis (CWP). We explored the baPWV in subjects withCWP and the associated risk factors.

**Methods:**

Thiscase-control study included 1,007 male CWP cases without a history of stroke and coronary heart disease and 1,007 matched controls from the Kailuan cohort study. All of the participants underwent assessment for baPWV and traditional cardiovascular risk factors. The cumulative silica dust exposure (work history linked to a job-exposure matrix) was estimated for the CWP cases.

**Results:**

Compared with the controls, the CWP cases had higher baPWV (1762.0 ± 355 cm/s vs. 1718.6 ± 354 cm/s, *P* = 0.006) and a higher risk of increased baPWV (defined as more than the median baPWV of the population distribution; odds ratio 1.43, 95% confidence interval 1.11–1.83) after adjusting for traditional cardiovascular risk factors. Age ≥60 years, body mass index, heart rate, and hypertension were all significantly associated with increased baPWV in the CWP cases. Compared to non-CWP subjects without hypertension, the odds ratios for increased baPWV gradually increased (P for trend, 0.001) across the CWP subjects without hypertension (odds ratio 1.20, 95%confidence interval 0.90–1.61), subjects with hypertension alone (odds ratio 2.54, 95% confidence interval 1.95–3.30), and CWP subjects with hypertension (odds ratio 3.34, 95% confidence interval 2.56–4.37). We detected a significant positive exposure-response relationship between silica dust-exposure quartiles and increased baPWV in CWP cases (P for trend < 0.001).

**Conclusions:**

For patients with CWP, increased baPWV was associated with traditional cardiovascular risk factors and long-term silica dust exposure.

## Background

Coal workers’ pneumoconiosis (CWP) is a chronic occupational lung disease caused by long-term inhalation of coal dust. Given that CWP is the most common occupational disease in coal workers [1–3], the prevalence of CWP is usually higher in developing countries than in developed countries. In China, the prevalence of CWP has been previously reported to be 6.02% (95% confidence interval (CI) 3.43–9.26%), which is a rate that is higher than those reported for the United Kingdom (0.8% for 1998-2000) and the United States (3.2% for the 2000s) [4–6].

Most cases of CWP are caused by silica exposure. Adverse health effects from long-term, cumulative silica dust exposure (CDE) are an increasing public health concern worldwide. Recent studies have reported that long-term CDE increased the risk of death not only due to respiratory diseases but also due to cardiovascular diseases (CVDs), revealing the exposure-response relationships between cumulative silica dust exposure and mortality from CVDs [7, 8]. These findings have increased the need to reduce the risk of cardiovascular mortality among patients with CWP. Since many CVDs can be prevented, early detection and prevention are key, particularly at the earlier stages of atherosclerosis. However, to our knowledge, no related research has been conducted among patients with CWP.

Arterial stiffness plays a critical role in the pathogenesis of atherosclerosis and cardiovascular events, is an independent predictor of cardiovascular mortality, and is a useful index in the prevention and early detection of CVD [9, 10]. Among representative measures of arterial stiffness, brachial-ankle pulse wave velocity (baPWV), which reflects the stiffness of both the central and peripheral muscular arteries, has been frequently used as a simple and non-invasive measure of systemic arterial stiffness [11–13]. Moreover, many studies have demonstrated that baPWV is an independent predictor of cardiovascular events [14] and mortality in the general population and in patients with an increased risk of CVDs [15–17]. Although baPWV is a useful tool for identifying subpopulations at increased risk for CVD, no observational data have been reported that indicate whether increased baPWV is associated with CWP and whether potential risk factors for increased baPWV occur among patients with CWP. Therefore, the present study sought to explore these two issues in Chinese patients with CWP.

## Methods

### Study population

The present case-control study included participants with CWP from the Kailuan cohort study, which recruited 101,510 employees and retirees of the Kailuan (Group) Co. Ltd., a large coal company located in Tangshan City, Hebei province, China, from June 2006 to October 2007. Details of this prospective cohort study were described previously [18–20]. The study followed the guidelines of the Helsinki Declaration and was approved by the Ethics Committees of Kailuan General Hospital and Beijing Chaoyang Hospital, China. Written informed consent was obtained from all the participants.

A total of 16,185 coal workers were included at baseline and followed up with an examination for pneumoconiosis every 2–3 years. As of December 31, 2010, 1,806 cases of CWP were diagnosed, which were all in male subjects. Some subjects were excluded from further analysis due to a failure to participate in the 2010–2011 resurvey due to limitations of activity (*n* = 441), death during the 2010–2011 resurvey (*n* = 123), age greater than 90 years (*n* = 22), refusal to undergo baPWV measurements (*n* = 166), incomplete baPWV data (*n* = 14), or a history of stroke, transient ischaemic attack, and/or coronary disease (*n* = 33). The present study therefore included 1,007 cases with CWP. This investigation also included 1,007 healthy controls from the Kailuan cohort study, all of which lacked a history of stroke, transient ischaemic attack, and coronary disease. These controls were matched to the cases based on age (±1 year), gender, systolic blood pressure (±5 mmHg), and previous history of hypertension.

### Diagnosis of pneumoconiosis

All of the enrolled CWP cases had physical examination cards and detailed records of their occupational history including CDE, individual medical and CWP diagnosis records, and measurements of dust concentrations in the subjects’ workplaces, which were obtained from personnel files in the human resources section of the Kailuan Colliery Group. The diagnosis of CWP was based on the Diagnostic Criteria of Pneumoconiosis and corresponding standard videos of pneumoconiosis in China [21]. CWP was classified as stage I, stage II, or stage III according to the size, profusion, and distribution range of opacities, as previously reported [22].

### Dust exposure data

Estimates of CDE were derived from each coal miner’s work history up until the time of study enrolment. Work histories included job titles and calendar years for each coal worker’s full duration of employment. CDE was calculated from a job-exposure matrix as follows: the duration of exposure in years was multiplied by the dust concentration at the same time in every period of dust exposure for each subject [22]. CDE is given in milligrams per cubic metre-years. Job title-specific exposure estimates were obtained from the Department of Dust Detection and Monitoring of the Kailuan Colliery Group. Dust was sampled randomly twice per month in the tunnelling, mining, combining, and helping areas. The dust concentration and free silica content were measured using the gravimetric method and the pyrophosphate method, respectively, which are national standard methods [23–25]. These numerical data were collected to calculate the geometric means of each area yearly, which were then used to calculate the CDE for each coal worker.

### Traditional cardiovascular risk factors

During the resurvey in 2010–2011, all of the participants underwent a clinical examination, laboratory tests, and baPWV measurements. Structured interviews based on a standardized questionnaire were conducted by trained investigators. The questionnaire included information on the subject’s demographics, history of occupational exposure, medical disorders, and traditional cardiovascular risk factors including age, smoking, body mass index, hypertension, diabetes and dyslipidaemia. Body mass index was calculated as body weight (kg) divided by the square of body height (m^2^). Current smokers were defined as subjects who had smoked at least 100 cigarettes during their lifetime and, at the time of the interview, reported smoking every day or some days.

### Measurement of BaPWV

In 2010–2011, all of the participants underwent baPWV measurements using an automatic arteriosclerosis detection device (BP-203RPE III; Omron Healthcare Co., Japan) in the supine position after at least 5 min of rest. BaPWV was calculated as the distance between the two sites divided by the pulse transit time, which was defined as the time interval between the wave front of the brachial waveform and that of the ankle waveform. The distance between the sampling points was calculated automatically according to the subject’s height. The maximum value of the bilateral baPWV was used in the present analysis.

### Laboratory measurements

Blood samples were obtained after at least 8 h of fasting and were analysed within 4 h. Fasting blood glucose levels were measured using the hexokinase/glucose-6-phosphate-dehydrogenase method. Total cholesterol and triglycerides were measured enzymatically (inter-assay coefficient of variation, 10%; Mind Bioengineering Co. Ltd., Shanghai, China). All biochemical variables were measured using an auto-analyser (Hitachi 747; Hitachi, Tokyo, Japan) at the central laboratory of Kailuan General Hospital.

### Statistical analyses

Statistical analyses were carried out using commercially available software (SAS software version 9.3; SAS Institute Inc., Cary, NC, USA). The continuous variables were described as the mean ± standard deviation and compared using a two-sample Student’s *t*-test or one-way analysis of variance for the normally distributed data. For skewed distributions, the data are presented as the median (with interquartile ranges) and compared using a Student’s *t*-test or one-way analysis of variance after log transformation. The categorical variables were described as percentages and compared using the chi-squared test. All tests were two-tailed and *P* < 0.05 was considered to be statistically significant.

Multivariate logistic regression analyses were used to explore the association of increased baPWV with CWP and its potential risk factors after adjusting for potential confounding factors. Increased baPWV was defined as a value greater than the median baPWV of the study population. Based on similar multivariate adjustments, the odds ratios (ORs) for increased baPWV were calculated for four subject subgroups: without hypertension or CWP, with CWP only, with hypertension only, and with hypertension and CWP. Similar analyses of the relationship between increased baPWV and CDE were conducted among the CWP cases categorized into four subgroups according to the CDE quartile using the trend test.

## Results

### Basic characteristic comparisons

The demographic and clinical characteristics of the CWP cases and healthy controls are presented in Table 1. All of the participants were male, and the mean age of the CWP cases was 65.2 years, which was similar to that of the controls (*P* = 0.853). There were no significant between-group differences in body mass index (*P* = 0.134), systolic and diastolic blood pressure (*P* = 0.508 and *P* = 0.108, respectively), low-density lipoprotein cholesterol (*P* = 0.640), and hypertension (*P* = 0.646). The CWP cases had higher heart rates (*P* < 0.001), fasting blood glucose levels (*P* = 0.003), and triglyceride levels (*P* = 0.001), but lower total cholesterol (*P* < 0.001) and high-density lipoprotein cholesterol (*P* = 0.033) levels than the controls. Significantly fewer CWP cases were smokers (*P* < 0.001) and they were more likely to have diabetes mellitus (*P* = 0.051) than the controls. The CWP cases were categorized as follows: stage I, 980 cases (97.3%); stage II, 22 cases (2.2%); and stage III, 5 cases (0.5%). The CWP cases had higher baPWV values than the controls (*P* = 0.006) and a higher proportion of the CWP cases (*P* = 0.016) had increased baPWV (≥1687 cm/s according to the median baPWV of the population) than the controls (Table 1).

**Table 1:** Demographic and clinical characteristics among CWP cases and controls

	CPW	Controls	*P*
N	1007	1007	
Age (Years)	65.15 ± 8.86	65.07 ± 8.82	0.853
Current smoking, n (%)	339 (33.7)	469(46.6)	<0.001
Body Mass Index (kg/m^2^)	25.41 ± 3.46	25.10 ± 3.97	0.134
Systolic Blood Pressure (mmHg)	138.78 ± 18.78	138.23 ± 18.94	0.508
Diastolic Blood Pressure (mmHg)	85.63 ± 10.91	84.84 ± 11.04	0.108
Heart rate (beats/min)	75.62 ± 16.39	71.31 ± 11.28	<0.001
Fasting Plasma Glucose (mmol/L)	5.89 ± 1.74	5.68 ± 1.39	0.003
Total Cholesterol (mmol/L)	4.91 ± 0.98	5.07 ± 1.00	<0.001
Low-Density Lipoprotein-Cholesterol (mmol/L)	2.64 ± 0.93	2.67 ± 1.00	0.640
High-Density Lipoprotein - Cholesterol (mmol/L)	1.52 ± 0.48	1.60 ± 1.06	0.033
Triglycerides (mmol/L)^a^	1.35 (1.02,1.81)	1.20 (0.86,1.70)	<0.001
Hypertension^b^, n (%)	612 (60.8)	612(60.8)	0.646
Diabetes mellitus^c^, n (%)	141(14.0)	111(11.0)	0.051
Stage			
I	980 (97.3)	-	
II	22 (2.2)	-	
III	5 (0.5)	-	
Duration of silica dust exposure (years)			
< 20	194 (19.3)		
20 ~ 29	447 (44.4)		
≥ 30	366 (36.3)		
Cumulative silica dust exposure (mg/m^3^-y)	1059.02 ± 458	-	
baPWV cm/s	1762.0 ± 355	1718.6 ± 354	0.006
baPWV ≥ 1687 cm/s, n (%)	533 (52.9)	478(47.5)	0.016

CWP indicates coal workers’ pneumoconiosis; baPWV, brachial-ankle pulse wave velocity

^a^Student’s *t*-test was used after log transformation

^b^Be defined as having the history of hypertension or SBP ≥ 140 mmHg or DBP ≥ 90 mmHg measured at the examination

^c^Be defined as having the history of diabetes mellitus or fasting blood glucose ≥ 7.0 mmol/L measured at the examination

### The association of BaPWV with CWP and traditional cardiovascular risk factors

In the logistic regression models shown in Table 2, the CWP cases had a higher risk of increased baPWV than the controls OR 1.24 (95% CI 1.05–1.48). After the multivariate adjustment for age, current smoking status, body mass index, heart rate, hypertension, diabetes mellitus, total cholesterol, and low-density lipoprotein cholesterol, the association remained significant OR 1.43 (95% CI 1.11–1.83).

**Table 2:** Logistic regression analyses of the associations of increased baPWV (≥1687 cm/s) with CWP and traditional cardiovascular risk factors among CWP cases and controls

	Univariate model		Multivariate model	
	OR (95% CI)	*P*	OR (95%CI)	*P*
Controls	1		1	
CWP	1.24 (1.05 - 1.48)	0.014	1.43(1.11 - 1.83)	0.005
Age > =60 years (yes vs no)	3.48(2.87-4.22)	<0.001	3.39(2.62 - 4.30)	<0.001
Current smoking (yes vs no)	0.78(0.66-0.94)	0.007	0.96(0.76 - 1.22)	0.760
Body Mass Index (kg/m^2^)	0.97(0.95-1.00)	0.065	0.95(0.92 - 0.98)	0.002
Heart rate (beats/min)	1.03(1.02-1.03)	<0.001	1.03(1.02 - 1.04)	<0.001
Hypertension (yes vs no)^a^	2.64(2.20-3.18)	<0.001	2.32(1.82 - 2.95)	<0.001
Diabetes mellitus (yes vs no)^b^	1.94(1.47-2.55)	<0.001	1.63(1.15 - 2.31)	0.006
Total Cholesterol (mmol/L)	1.08(0.99-1.18)	0.102	1.11(0.98 - 1.27)	0.106
Low-Density Lipoprotein-Cholesterol (mmol/L)	0.99(0.91-1.09)	0.895	0.94(0.82 -1.07)	0.335

CWP indicates coal workers’ pneumoconiosis; baPWV, brachial-ankle pulse wave velocity

^a^Be defined as having the history of hypertension or SBP ≥ 140 mmHg or DBP ≥90 mmHg measured at the examination

^b^Be defined as having the history of diabetes mellitus or fasting blood glucose ≥7.0 mmol/L measured at the examination

A further stratification analysis of the potential risk factors for baPWV (Table 3) showed that in both the CWP and control groups, age (≥60 years), heart rate, and hypertension were positively associated with increased baPWV (all *P* < 0.001). In addition, increased baPWV was also positively associated with diabetes mellitus (*P* = 0.012) and negatively associated with current smoking status (*P* < 0.001) in the control group.

**Table 3:** Multivariate logistic regression analyses of the associations of increased baPWV (≥1687 cm/s) and traditional cardiovascular risk factors among CWP cases and controls

	CWP *n* = 1007		Controls *n* = 1007	
	OR (95% CI)	*P*	OR (95%CI)	*P*
Age > =60 years (yes vs no)	3.12(2.39-4.09)	<0.001	3.92(2.96-5.19)	<0.001
Body Mass Index (kg/m^2^)	0.97(0.93-1.00)	0.062	0.98(0.94-1.03)	0.426
Heart rate (beats/min)	1.03(1.02-1.04)	<0.001	1.03(1.02-1.04)	<0.001
Hypertension (yes vs no)^a^	2.78(2.14-3.61)	<0.001	2.54(1.95-3.30)	<0.001
Diabetes mellitus (yes vs no)^b^	1.37(0.95-1.97)	0.089	2.93(1.91-4.50)	<0.012
Current smoking (yes vs no)	1.01(0.78-1.31)	0.939	0.65(0.51-0.84)	<0.001
Total Cholesterol (mmol/L)	1.05(0.92-1.20)	0.452	1.12(0.99-1.27)	0.070
Low-Density Lipoprotein-Cholesterol (mmol/L)	0.94(0.82-1.08)	0.355	1.05(0.92-1.19)	0.493

CWP indicates coal workers’ pneumoconiosis; baPWV, brachial-ankle pulse wave velocity

^a^Be defined as having the history of hypertension or SBP ≥ 140 mmHg or DBP ≥ 90 mmHg measured at the examination

^b^Be defined as having the history of diabetes mellitus or fasting blood glucose ≥ 7.0 mmol/L measured at the examination

### The combined effects of CWP and hypertension on the BaPWV

All of the participants were categorized into four subgroups (Table 4), and the subgroup of subjects without CWP or hypertension was employed as a reference in the subsequent calculations of the ORs of increased baPWV. We detected a gradually increasing association strength across the four subgroups (P for trend <0.001) as follows: CWP only, OR 1.20 (95% CI 0.90–1.61); hypertension only, OR 2.54 (95% CI 1.95–3.30); and both CWP and hypertension, OR 3.34 (95% CI 2.56–4.37). The trend persisted after the multivariate adjustment (P for trend, <0.001), and the multivariate-adjusted ORs were 1.18 (95% CI 0.85–1.16), 1.55 (95% CI 1.92–3.41), and 2.80 (95% CI 2.08–3.75).

**Table 4:** Unadjusted and multivariate adjusted odds ratio (95% CI) for increased baPWV (≥1687 cm/s) among participants with CWP and/or hypertension and without (*n* = 2014) CWP indicates coal workers’ pneumoconiosis; baPWV, brachial-ankle pulse wave velocity

	Unadjusted OR (95% CI)	*P*	Multivariate adjusted OR (95% CI)^a^	*P*
Without CWP and hypertension	1		1	
With only CWP	1.20 (0.90 - 1.61)	0.219	1.18 (0.85 - 1.16)	0.313
With Only hypertension	2.54 (1.95 - 3.30)	<0.001	1.55 (1.92 - 3.41)	<0.001
With CWP and hypertension	3.34 (2.56 - 4.37)	<0.001	2.80 (2.08 - 3.75)	<0.001
*p*_trend_	<0.001		<0.001	

^a^Adjustment for age (≥60 years), current smoking, body mass index, diabetes mellitus, heart rate, total cholesterol, low-density lipoprotein-cholesterol

### The exposure-response relationship between CDE and BaPWV

**Fig. 1:**
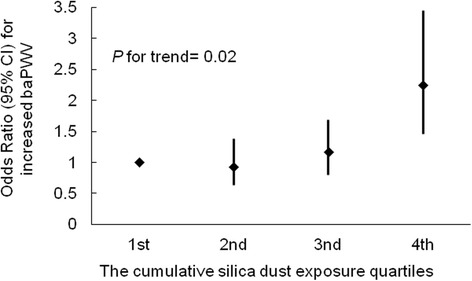
Mulitvariate Logistic regression analyses of the associations of increased baPWV with cumulative dust exposure (CDE) quartiles among CWP cases. The level of CDE quartiles in all CWP cases was as follow: first,≤811 mg/m^3^-y; second, 811-1059 mg/m^3^-y, third, 1059-1320 mg/m^3^-y, and fourth, ≥1320 mg/m^3^-y

To explore the association between long-term CDE and increased baPWV (Fig. 1), all of the CWP cases were categorized into four subgroups according to the CDE quartile. We observed that the risk of increased baPWV gradually increased across the CDE quartiles, independent of traditional cardiovascular risk factors. The multivariate-adjusted ORs (compared with the cases in the first quartile) for the cases in the second, third, and fourth CDE quartiles were 1.02 (95% CI 0.61–1.70), 1.40 (95% CI 0.87–2.24), and 2.23 (95% CI 1.30–3.83), respectively. This trend was statistically significant (P for trend, 0.02). The adjusted variables included age, current smoking status, body mass index, heart rate, hypertension, diabetes mellitus, total cholesterol, and low-density lipoprotein cholesterol.

## Discussion

Mortality due to CVD among coal workers with CWP is increasing [7, 8], which highlights the pressing need to tackle this health challenge. Early detection of CVD is key. Early detection of arterial stiffness via measurement of baPWV is useful in primary and secondary prevention of CVDs such as hypertension and coronary artery disease. Several studies have shown that baPWV is an independent predictor of the future development of CVD [7]. To the best of our knowledge, this investigation is the first demonstration of a relationship between baPWV and CWP in a relatively large sample of subjects without a history of symptomatic stroke, transient ischaemic attack, or coronary disease.

Here, the CWP cases had higher baPWV values than the controls, as well as a higher risk of increased baPWV. This risk was independent of traditional cardiovascular risk factors, including age, current smoking status, body mass index, hypertension, diabetes mellitus, total cholesterol, and low-density lipoprotein cholesterol. In this population, increased baPWV may be an independent and novel complication of CDE.

Among the CWP cases, the risk factors for baPWV included several traditional cardiovascular risk factors, such as age (≥60 years), heart rate, and hypertension. In the healthy controls, baPWV was also significantly associated with diabetes mellitus. These findings agree with those of previous investigations of the general population and of patients who are at a higher risk of cardiovascular events. These investigations reported that baPWV increases with age, hypertension [26], increased heart rate [27], and diabetes [28].

However, in our multivariate analyses, no statistically significant associations of increased baPWV were found with total cholesterol and low-density lipoprotein-cholesterol, which might reflect selection bias in our case-control study. We enrolled a portion of the subjects from another large-scale cohort study and excluded the individuals with a history of stroke, transient ischaemic attack, and coronary diseases, which might conceal the associations that would be demonstrated in a longitudinal study. Similar reasons may explain the results of association of increased baPWV with current smoking. First, physicians would advise individuals with CWP to quit smoking for a better prognosis, which might lead to the lower prevalence of CWP cases who current smoke. As shown in Table 2, the proportion of current smoking in the CWP cases was lower than the controls (33.7% vs. 46.6%, *P* < 0.001). Second, our use of self-reported smoking status collected at the time of study enrolment, rather than a sensitive and specific marker of exposure to tobacco, such as cotinine, resulted in the introduction of misclassification bias into our study of smoking and baPWV. The potential for these biases should be considered when interpreting the negative association between smoking and baPWV among the controls and as well as the lack of a statistically significant association among the CWP workers.

Hypertension is a leading risk factor for CVD. Its prevalence is high and has increased rapidly over the past four decades in China, from 5% in 1959 to 18% in 2002 [29, 30]. In the present study, hypertension occurred more frequently in the CWP cases (60%), and of the identified risk factors, hypertension had a relatively stronger effect on baPWV. Based on these considerations, we further evaluated the combined effects of CWP and hypertension on baPWV, and showed that the combined effect of these parameters increased baPWV more than either factor alone. Future studies of patients with CWP should be conducted to determine the extent to which the prevention and control of disease progression, combined with appropriate anti-hypertensive medications, can stabilize or even reverse arterial stiffness.

More importantly, the present study revealed an exposure-response relationship between CDE and baPWV in the CWP cases, which was independent of traditional cardiovascular risk factors. No previous reports have investigated this association. Large-scale cohort studies previously uncovered an exposure-response relationship between CDE and increased mortality from CVDs [7, 8]. On the basis of these findings, one might hypothesize that by increasing arterial stiffness, long-term CDE increases the risk of cardiovascular events. This hypothesis should be tested in prospective cohort studies.

### The mechanisms by which long-term CDE might increase arterial stiffness, as measured by baPWV, are largely unknown

Inflammation is one plausible mechanism. Atherosclerosis, a pathological process of CVD, is now generally accepted to be an inflammatory disorder of the arterial wall [31]. Inflammation may also contribute to baPWV elevation. For example, Saijo et al. reported a significant, progressive increase in baPWV with high-sensitivity C-reactive protein levels in male subjects after controlling for traditional cardiovascular risk factors such as age, body mass index, systolic blood pressure, heart rate, smoking, past history of hypertension, hyperlipidaemia, and diabetes [32]. Andoh et al. also determined that baPWV is significantly associated with the serum levels of high-sensitivity C-reactive protein [33]. Moreover, CWP is caused by the long-term inhalation and deposition of coalmine dust, which triggers a persistent inflammatory response and the induction of pro-inflammatory and pro-fibrotic mediators, which eventually results in irreversible lung damage [34, 35]. Respirable silica particles can initiate inflammation of the cardiovascular system via the direct effects of fine particulates that cross the pulmonary epithelium into the cardiovascular system [36] or via indirect effects mediated by the inflammatory response.

Evidence of the association between increased systemic inflammation and CWP is limited. Zhai et al. reported that the serum levels of cytokines such as interleukin 6 were associated with CWP in a Chinese sample [37]. Lee et al. suggested that high serum levels of interleukin 8 in Korean subjects were associated with CWP and those of serum tumour necrosis factor α were associated with the progression of CWP at the 1-year follow-up [38], but not at the 3-year follow-up [39]. In addition, studies of Chinese CWP cases revealed associations between CWP and genetic polymorphisms related to inflammatory markers such as E-selectin [40] or the inflammasome (nod-like receptor protein 3) [41]. Combining these previous data with the findings of the current investigation, it is logical to hypothesize that long-term CDE can instigate the inflammation response and damage arterial walls, which leads to atherosclerosis and cardiovascular events. This hypothesis needs to be validated using large-scale prospective cohort studies.

Several limitations of this study should be considered when interpreting the data. First, in light of the small sample size of the participants with CWP in Stage II and III (2.2% and 0.3%, respectively), we could not explore the association of the severity of CWP with baPWV. Second, we were unable to elucidate the role of inflammation in the observed relationship between CWP and baPWV. Third, increased arterial stiffness is thought to increase the risk of CVD; however, baPWV only reflects the stiffness of middle-sized to large arteries and is closely correlated with carotid-femoral pulse wave velocity, which is a gold standard for the assessment of large-artery stiffness. Most importantly, the case-control design of our study hindered us from evaluating the prognostic significance of increased baPWV in CWP cases. In addition, coal dust is a mixture and other components such as polycyclic aromatic hydrocarbons might have cardiovascular effects. It is also possible that other co-exposures in the mines might contribute to the development of cardiovascular diseases. Therefore, longitudinal studies of factors that affect cardiovascular prognosis as well as studies investigating the exposure to the components of coal dust and other co-exposures in the mines are necessary in order to establish a better understanding of the risk factors of arterial stiffness and cardiovascular risk in patients with CWP.

## Conclusion

In conclusion, in the present study of more than 2,000 participants, CWP was significantly associated with baPWV, independent of additional risk factors for baPWV. The combined effect of CWP and hypertension on baPWV was stronger than either factor alone. Moreover, we uncovered an exposure-response relationship between CDE and the risk of increased baPWV. The prognostic value of baPWV for the incidence of cardiovascular events among patients with CWP should be analysed in future prospective studies.
